# Attention-deficit/hyperactivity disorder Under Treatment Outcomes Research (AUTOR): a European observational study in pediatric subjects

**DOI:** 10.1007/s12402-015-0177-y

**Published:** 2015-06-27

**Authors:** Virginia Haynes, Pedro Lopez-Romero, Ernie Anand

**Affiliations:** Lilly Corporate Center, Eli Lilly and Company, Indianapolis, IN 46285 USA; Eli Lilly and Company, Madrid, Spain; Eli Lilly and Company, Erl Wood, UK

**Keywords:** Attention-deficit/hyperactivity disorder, Atomoxetine, ADHD, Quality of life, Treatment, Observational

## Abstract

The ADHD Under Treatment Observational Research (AUTOR) study was a European prospective, observational study that assessed factors associated with changes in ADHD severity, estimated change from baseline in quality of life (QoL), and characterized changes in ADHD symptoms over a 2-year period as a function of baseline treatment. The primary objective was to identify factors associated with worsening in ADHD severity during a 2-year follow-up period for subjects aged 6–17 years, who were receiving the same pharmacotherapy for 3–8 months before enrollment and had a Clinical Global Impression (CGI)-ADHD-Severity score of mild/lower and a CGI-ADHD-Improvement score of improved/very much improved. Multivariate logistic regression examined the association of factors with worsening in ADHD. Mixed-model repeated measures regression analyzed QoL in terms of change from baseline in CHIP-CE PRF scores. There were 704 subjects analyzed. Variables associated with worsening ADHD severity were parental occupation, poorer school outcomes, and use of psychoeducation; baseline treatment was not significant. Among the secondary objectives, initial use of atomoxetine (vs. stimulants) was associated with a significant improvement on the CHIP-CE PRF total score, with an adjusted treatment difference of −6.0 (95 % CI −7.9, −4.1) at 24 months. Additionally, the odds of stability (CGI-ADHD-S ≤ 3 over the 2-year period) were significantly lower for subjects initially responding to stimulants compared with atomoxetine (OR 0.5; 95 % CI 0.3, 0.8). ADHD symptom worsening was associated with initial use of psychoeducation, parental occupation, and poorer school outcomes. Response to initial treatment with atomoxetine was associated with improved QoL over 2 years.

## Introduction

Attention-deficit/hyperactivity disorder (ADHD) is characterized by inattention, impulsivity, and hyperactivity and is associated with other psychiatric comorbidities (Pliszka 2000; Spencer 2006). Patients are at a higher risk of cigarette smoking, substance abuse (Daley 2004; Vansickel et al. 2007), more traffic accidents (Barkley 1998; Weiss et al. 1999), and criminality (Klein and Mannuzza 1991). Adverse consequences persist through adolescence into adulthood, including academic impairment, social dysfunction, poor self-esteem (Biederman et al. 2004), and increased rates of suicide (Barbaresi et al. 2013). Thus, the burden of illness associated with ADHD is high for affected individuals, their families, and society (Leibson and Long 2003).

Current treatments for ADHD include social, psychological, and behavioral interventions and pharmacotherapy. The efficacy of pharmacotherapy for ADHD has been investigated in well-documented, short-term studies (Biederman et al. 2004; Perwien et al. 2004). An important question is whether there is a continued value of ADHD pharmacotherapy treatment for subjects who had an initial clinical response. Children and adolescents who responded to open-label atomoxetine treatment maintained their responses for up to 18 months (Michelson et al. 2004; Buitelaar et al. 2007). Maintenance of response to atomoxetine in adults for up to 6 months has also been demonstrated (Upadhyaya et al. 2013).

Most research on the efficacy of ADHD treatments has been conducted within a clinical trial setting in which subject eligibility is restricted by a large set of inclusion and exclusion criteria. In clinical practice, however, the subject population receiving ADHD medications has a wider array of clinical comorbidities than allowed in most clinical trials. Subjects may be prescribed various types of treatment concomitantly, and these treatments may change over time. Thus, it would seem that real-world outcomes would be of considerable interest, but few naturalistic/observational studies have been conducted in pediatric subjects with ADHD.

The Attention-Deficit/Hyperactivity Disorder Observational Research in Europe (ADORE) study, a 24-month, observational study of approximately 1500 children and adolescents in 10 European countries, analyzed long-term treatment patterns (pharmacotherapy, psychotherapy, combination of both, or no intervention) for ADHD and the associated health outcomes for subjects following their diagnosis and first treatment in European naturalistic practice settings (Preuss et al. 2006). Subjects in the ADORE pharmacotherapy treatment groups showed greater improvement than those with nonpharmacotherapy. This finding was similar to that of the Multimodal Treatment Study of ADHD (MTA) (Jensen et al. 2007), a randomized clinical study aimed at comparing the effects of medication management, behavior therapy, a combination of medication and behavior therapies, and usual community care over a 14-month period in the USA (The MTA Cooperative Group 1999), with noninterventional follow-ups at 3 and 8 years (Jensen et al. 2007; Molina et al. 2009). Neither the MTA nor the ADORE study assessed the changes in symptomatology after response to treatment; rather, the focus of these studies was to follow-up with subjects after their initial treatment to determine whether that treatment was effective or ineffective. The AUTOR study was designed to augment the findings of earlier maintenance clinical trials (Gillberg et al. 1997; Michelson et al. 2004; Buitelaar et al. 2007) by characterizing longer-term treatment patterns among pharmacotherapy responders and factors associated with loss of response in the clinical practice setting across Europe.

The AUTOR study is a longitudinal, observational, naturalistic study conducted in subjects from 6 to 17 years old who are diagnosed with ADHD. The primary objective was to identify the factors associated with an increase in ADHD symptom severity during a 2-year follow-up period in subjects who were responders and were stable on their initial pharmacotherapy. Secondary objectives related to effectiveness and tolerability were to describe the factors associated with a decrease in ADHD symptom severity, quality of life (QoL) changes, treatment patterns, factors associated with relapse, factors associated with stability, tolerability, and the duration of treatment effect during the day associated with different pharmacotherapies.

## Materials and methods

As the primary objective, characterizing factors associated with worsening in ADHD symptoms, was associated with a binary endpoint of worsening or no worsening, the study protocol approximated a sample size of 900 patients using a two-sided Chi-square test at a 5 % significance level in order to have a 80 % power to detect a difference between group 1 proportion p1 = 0.5 and group 2 proportion p2 = 0.59. Slower than expected enrollment resulted in 704 patients in the analysis set, causing only a slight increase in the minimum difference that could be detected between the two group proportions (p1 = 0.5 and p2 = 0.6).

### Subjects

Physicians enrolled patients aged 6–17 years old who had been diagnosed with ADHD and who had responded to their first and current pharmacotherapy treatment for 3–8 months. Clinical responders were identified as having a Clinical Global Impression-ADHD-Severity (CGI-ADHD-S) score of mild or lower (CGI-ADHD-S ≤ 3 at study entry) and a Clinical Global Impression-ADHD-Improvement score (CGI-ADHD-I) of much improved or very much improved (CGI-ADHD-I = 1 or 2) (Arnold et al. 2004) at the baseline observation compared to the time of treatment initiation. Subjects were excluded if they had, in the clinical judgment of the investigator, a pervasive developmental disorder or were already participating in another treatment study.

The requirement for treatment stability at study entry increased the naturalistic character of the trial by ensuring that treatment patterns were not altered due to participation in the study. During AUTOR, subjects were allowed to take any commercially available medication (including combination therapy) or nonpharmacotherapy for the treatment of ADHD; ADHD treatment could be discontinued or changed at any time, and subjects were followed in the study, regardless of changes or discontinuation of their original ADHD treatment.

Subjects were informed as to the risks and benefits of trial participation; their parents gave written consent and they provided written assent for the use of their data, as required by local regulations. The study was conducted in accordance with the ethical principles of the Declaration of Helsinki and was consistent with good clinical practices and applicable local laws and regulations.

### Procedure

Data were collected at naturally occurring visits for the subjects, according to regular practice at the study site; these visits were assigned to the closest of the following observation windows: 0, (baseline), 3, 6, 9, 12, 18, and 24 months (from baseline), ± 6 weeks. Apart from baseline confirmation of eligibility and capture of subject/family information and demographics, all other assessments were performed at each visit.

Symptom severity was measured with the CGI-ADHD-S and the ADHD Rating Scale-IV-Parent Version-Investigator-completed (ADHDRS). ADHD symptom severity worsening was defined as a ≥2-point increase from baseline in CGI-ADHD-S score. A two-point worsening on CGI-ADHD-S was included to identify clinically meaningful relapse in the relapse prevention study of atomoxetine (Michelson et al. 2004) and a lisdexamphetamine trial (Coghill et al. 2014). A decrease in ADHD severity was defined as a ≥2-point decrease in the CGI-ADHD-S from one of the follow-up observations to any of the subsequent observations.

Information on the use of pharmacotherapy and other treatments for ADHD was collected at each visit. Pharmacotherapy treatment classes at baseline were a priori defined for analysis as stimulant, atomoxetine, other, and combination. The combination class comprised subjects taking more than one pharmacotherapy class. Subjects in any treatment class could receive nonpharmacotherapy sessions. A change in pharmacotherapy was defined as moving from one class to another or changing to only nonpharmacotherapy treatment. Discontinuation from therapy was defined as no pharmacotherapy and no nonpharmacotherapy for at least 4 weeks.

QoL changes over the 2-year period were measured by the Child Health and Illness Profile, Child Edition-Parent Report Form (CHIP-CE PRF) (Riley et al. 2001, 2004).

Four different definitions of relapse were used: (1) increase of 50 % or greater on the ADHDRS total score and an increase in the CGI-ADHD-S score of at least 2 points, (2) increase of at least 50 % on the ADHDRS total score from the baseline, (3) a CGI-ADHD-S score of at least markedly ill (≥5) at any post-baseline assessment, and (4) a minimum of a 2-point increase on the CGI-ADHD-S from baseline over 2 consecutive post-baseline assessments. Stability was defined as CGI-ADHD-S of “mild” or lower (≤3) over the entire 2-year period. Relapse definition 1 was used in the pivotal maintenance of response study of atomoxetine (Michelson et al. 2004) and a recent lisdexamphetamine maintenance of response trial (Coghill et al. 2014). Relapse definition 2 included the ADHDRS symptom assessment only, and relapse definition 4 required a repeated observation of worsening to flag relapse.

Duration of treatment effect during the day associated with different pharmacotherapies was measured by the Global Impression of Perceived Difficulties (GIPD) scale (Wehmeier et al. 2008).

### Statistical analysis

#### General considerations

Analyses were exploratory. Two baseline treatment classes (stimulant and atomoxetine) were compared with respect to their effect on worsening of ADHD severity and other secondary outcome/tolerability measures. All statistical analyses were pre-specified in a Statistical Analysis Plan that was approved before database lock. All statistical analyses were performed using SAS version 9.2 (SAS Institute, Inc., Cary, NC, USA). No corrections were made for multiple comparisons.

#### Demographics and treatment compliance and patterns

Descriptive statistics were used to summarize subject characteristics, total daily dose by baseline pharmacological treatment group and by time point, compliance by baseline pharmacological treatment group, number of sessions per month by baseline nonpharmacological treatment and time point, and time to first change/switch/discontinuation of therapy. The Kaplan–Meier method was used to estimate the survival curves for time to first change/switch/discontinuation of therapy and comparison between treatment groups were conducted by a 2-sided log-rank test. Subjects discontinuing the study without discontinuing treatment were considered censored at the time of exiting the study.

#### Primary outcome measure

Multivariate logistic regression was used to identify factors associated with worsening in ADHD severity. A patient was considered to have a worsening in ADHD severity if a minimum of two points increase in the CGI-ADHD-S score (vs. baseline CGI-ADHD-S score) was observed at any of the subsequent follow-up observations. The set of covariates are listed in Table 1. Covariates that were noncorrelated (*r* < 0.7) and statistically significant in univariate logistic regression models (*p* < 0.10) were included in the full multivariate logistic models. The analysis plan allowed for treatment to be included as a time-varying covariate only if >25 % of patients switched their baseline medication; however, the proportion of patients switching treatments was much lower so treatment was not included as a time-varying covariate. Treatment compliance was included as a time-varying covariate in the multivariate models; however, the addition of this covariate did not change the model estimates and was dropped from the final model. In addition, propensity scores (PS), estimated using multivariable logistic regression, were included in the models as additional covariates to adjust for the probability of receiving a specific treatment given the subject gender, age, ADHD subtype, family history of ADHD, substance use, psychiatric comorbidities and resource utilization baseline variables. Logistic models with and without PS covariates were estimated. For the final multivariable model, Type III *p* values and adjusted odds ratios comparing each level against an arbitrary baseline reference level and associated 95 % CI were calculated.

**Table 1:** Covariates considered in the logistic regression modeling process for primary and secondary endpoints

Independent variables	Categories	Reference level
Baseline covariates		
Gender	Male versus female	Female
Age	5–12 versus 13–18	13–18 years
ADHD subtype	Hyperactive/impulsive or combined, inattentive	Inattentive
Family history of ADHD	Immediate family, extended family, no family history	No family history
Substance use	Yes (if smoked, used alcohol, or used illegal drugs) versus no substance use	No substance use
Psychiatric comorbidities	Current presence of Tourette’s Disorder, tics, anxiety, depression, conduct disorder, oppositional defiant disorder, bipolar disorder, or psychosis versus no current presence of 1 of these conditions	No current presence of these comorbidities
Total number of contacts to all healthcare providers	Continuous	N/A
Origin	West Asian, East Asian, Hispanic, Black or African-American, White	White
Family setting	Child lives with single biological parent, guardian, biological parents separately, both biological parents, other	Other
Number of siblings	Continuous	N/A
Parental work status	Working for pay full-time, part-time work, unemployed, keeping house	Working for pay full-time
Psychoeducation	Yes, no	No
ADHDRS total score	Treated as continuous	N/A
Parental occupation	Elementary occupations; managers and senior officials; process, plant and machine operatives; sales and customer service; caring, leisure and other personal service; skilled trades; administrative and secretarial; associate professional and technical occupations	Managers and senior officials
School outcomes (in 3 months prior to study entry)	Not in school during the past 3 months, suspended from school, expelled from school, or requested to change to a special need school; Some exclusion from school lessons or in a special education program; or manageable in a classroom environment	Manageable in a classroom environment
Propensity score	Continuous	N/A
Treatment	Stimulant, atomoxetine	Atomoxetine
Time-varying covariates		
Compliance	Never, Always, Occasionally, Some of the Time, Most of the Time	Always

*N/A* nonapplicable, *vs.* versus

#### Secondary outcomes

Mixed models for repeated measures (MMRM) were used to estimate adjusted differences between stimulant and atomoxetine baseline treatments in relation to changes from baseline for GIPD total scores and items and the CHIP-CE PRF standardized total, domain, and subdomain scores. The MMRMs contained baseline treatment, visit, baseline treatment-by-visit and PS and baseline treatment-by-PS interactions as independent variables and used an unstructured covariance matrix to model the between-subjects and within-subjects errors. Other covariates considered in the model were age, gender, ADHD subtype, family history of ADHD, drug consumption, psychiatric comorbidities, score at baseline, compliance at baseline, tolerability at baseline, treatment satisfaction at baseline, school outcomes at baseline, baseline bullying, and total number of contacts to healthcare providers at baseline. Of these covariates, only covariates found to be significant (*p* < 0.10) in a first-step model (including baseline treatment, visit, baseline treatment-by-visit and PS, baseline treatment-by-PS interactions, and the covariate of interest) were retained in the final multivariable model. Multivariate logistic regression models were used to estimate adjusted odds ratios for variables associated with relapse and stability. The covariates were the same as those used in the analysis of the primary outcome variable (Table 1). Logistic regression models, with and without PS, and with and without treatment compliance as time-varying covariates, were estimated.

#### Tolerability

The number and percentage of subjects with solicited AEs were calculated for each visit within the treatment class at that visit. The effect of baseline treatment on AEs was assessed with a logistic regression model for repeated measures using a population-averaged GEE approach. GEE models included baseline treatment, visit, treatment-by-visit interaction, propensity score, and treatment-by-PS interactions as independent variables. An unstructured covariance matrix was used to model the between and within subject errors. For each AE, adjusted odds ratios between treatments at each visit and averaged over all visits were estimated.

## Results

### Disposition, demographics, and baseline characteristics

The AUTOR study was conducted at 74 study centers in Denmark, Greece, Italy, Netherlands, Romania, Slovenia, Sweden, and the UK. Subject enrollment began September 2008 and completed in February 2013. Practice settings (93 % urban) were 4 % inpatient, 47 % outpatient, and 49 % a combination of both and were 8 % private, 68 % public, and 24 % a combination of both.

The majority (86 %) of investigators were child psychiatrists, and the remainder were neurologists, child neurologists, and pediatricians with an average duration of practice of 25 years. Of the 801 subjects who entered the study, 704 met entry criteria and comprised the analysis set. At baseline, 704 subjects were stable on and responsive to the following medications: stimulants (*N* = 302 [48 % methylphenidate and 53 % methylphenidate long-acting]), atomoxetine (*N* = 395), other pharmacotherapies (*N* = 5 [60 % antipsychotics and 40 % other), or a drug combination (*N* = 2 [100 % methylphenidate long-acting and atomoxetine]) (Table 2).

**Table 2:** Patient flow from baseline to month eighteen

Analysis visit	Stimulant	Atomoxetine
Baseline	*N* = 302	*N* = 395
3 Months		
Discontinued at previous visits	10	5
Pharmacotherapy nonstimulant	N/A	376
Pharmacotherapy stimulant	272	N/A
Drug combination	1	1
Present at further visits, did not attend current visit	19	13
6 Months		
Discontinued at previous visits	14	18
Pharmacotherapy nonstimulant	2	342
Pharmacotherapy stimulant	256	3
Drug combination	1	2
Nonpharmacotherapy	1	4
No treatment for primary study condition	3	3
Present at further visits, did not attend current visit	25	23
9 Months		
Discontinued at previous visits	22	26
Pharmacotherapy nonstimulant	2	326
Pharmacotherapy stimulant	231	7
Drug combination	N/A	1
Nonpharmacotherapy	2	7
No treatment for primary study condition	7	11
Present at further visits, did not attend current visit	37	17
Discontinued after month 6	1	N/A
12 Months		
Discontinued at previous visits	32	37
Pharmacotherapy nonstimulant	8	309
Pharmacotherapy stimulant	231	12
Drug combination	2	5
Nonpharmacotherapy	3	8
No treatment for primary study condition	8	14
Present at further visits, did not attend current visit	16	9
Discontinued after month 6	1	N/A
Discontinued after month 9	N/A	1
Completed at previous visits	1	N/A
18 Months		
Discontinued at previous visits	44	42
Pharmacotherapy nonstimulant	8	293
Pharmacotherapy stimulant	217	15
Drug combination	2	3
Nonpharmacotherapy	3	12
No treatment for primary study condition	9	20
Present at further visits, did not attend current visit	17	9
Discontinued after month 6	1	N/A
Discontinued after month 9	N/A	1
Completed at previous visits	1	N/A
24 Months		
Discontinued at previous visits	67	62
Pharmacotherapy nonstimulant	9	265
Pharmacotherapy stimulant	206	17
Drug combination	1	6
Nonpharmacotherapy	3	12
No treatment for primary study condition	3	31
Discontinued after month 6	1	N/A
Discontinued after month 9	N/A	1
Discontinued after month 18	4	N/A
Completed at previous visits	8	1

*N* number of subjects, *N/A* not applicable

**Fig. 1:**
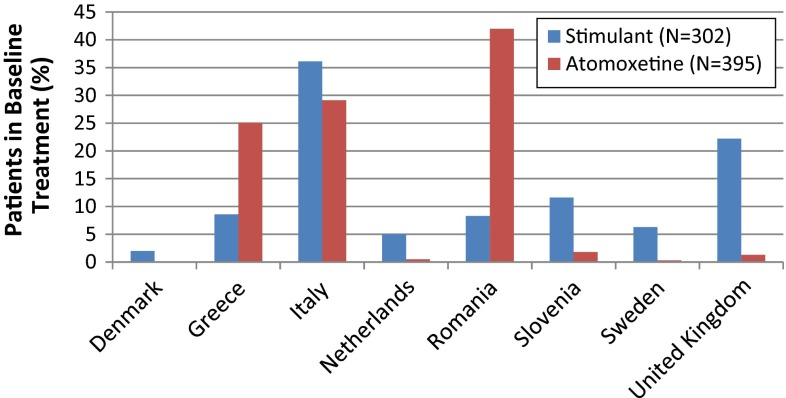
Countries participating in AUTOR

Of the 704 subjects in the study, the majority were Caucasian (98.9 %) and male (81.5 %). Nearly 80 % of the subjects completed the 2-year study. Table 3 summarizes the physical characteristics, comorbidities, prior treatment duration, and disposition of the sample. Figure 1 summarizes the subjects by country. The majority of subjects were recruited from Italy, Romania, and Greece, and the pattern of allocation to treatment reflects the timing of medication availability in those regions.

**Table 3:** Physical characteristics, psychiatric comorbid conditions, disposition, and prior treatment duration

Baseline treatment group	Stimulant *N* = 302	Atomoxetine *N* = 395
Physical characteristics, mean (SD)		
Age	10.9 (2.6)	10.6 (2.8)
Height	145.0 (16.6)	143.1 (17.2)
Weight	40.1 (15.2)	39.7 (15.8)
BMI	18.5 (3.6)	18.7 (3.9)
ADHD subtype		
Predominantly inattentive, *n* (%)	42 (13.9)	62 (15.7)
Predominantly hyperactive impulsive, *n* (%)	21 (7.0)	40 (10.1)
Combined, *n* (%)	239 (79.1)	293 (74.2)
ADHD severity		
CGI-ADHD-S, mean (SD)	2.6 (0.50)	2.5 (0.60)
ADHDRS total score, mean (SD)	25.1 (11.40)	22.8 (11.81)
ADHDRS Inattentive Subscale, mean (SD)	13.3 (6.07)	12.5 (6.00)
ADHDRS Hyperactivity-Impulsivity Subscale, mean (SD)	11.8 (6.28)	10.3 (6.82)
Psychiatric comorbidities, *n* (%)		
Anxiety	24 (7.9 %)	82 (20.9 %)
Depression	10 (3.3 %)	19 (4.8 %)
Conduct disorder	31 (10.3 %)	43 (10.9 %)
Oppositional defiant disorder	88 (29.1 %)	108 (27.5 %)
Tourette’s syndrome	1 (0.3 %)	1 (0.3 %)
Tics	13 (4.3 %)	22 (5.6 %)
Coordination problems	28 (9.3 %)	28 (7.1 %)
Dyslexia	51 (16.9 %)	89 (22.6 %)
Other learning disorders	90 (29.8 %)	137 (34.9 %)
Bipolar disorder	1 (0.3 %)	0 (0.0 %)
Psychosis	1 (0.3 %)	0 (0.0 %)
Obsessive compulsive disorder	2 (0.7 %)	3 (0.8 %)
Subject disposition, *n* (%)		
Completed	223 (73.8 %)	325 (82.3 %)
Discontinued	79 (26.2 %)	70 (17.7 %)
Caregiver decision	27 (8.9 %)	41 (10.4 %)
Loss to follow-up	26 (8.6 %)	15 (3.8 %)
Subject decision	15 (5.0 %)	12 (3.0 %)
Physician decision	11 (3.6 %)	2 (0.5 %)
Duration of baseline treatment (months), mean (SD)	4.9 (1.5)	5.0 (1.5)

*N* number of subjects, *n* number of affected subjects, *SD* standard deviation

### Treatment compliance and patterns

Treatment compliance was estimated by clinical staff at each visit by selecting for how they took medication—never, occasionally do, some of the time, most of the time, and always. Treatment compliance was comparable across baseline treatment groups, and it decreased throughout the study with the highest compliance observed at baseline (74.0 % always, 21.6 % most of the time, 2.3 % some of the time, 1.2 % occasionally do, and 0.9 % never) and the lowest compliance observed at Month 24 (65.1 % always, 26.1 % most of the time, 5.6 % some of the time, 1.8 % occasionally do, and 1.4 % never). The average total daily dose of medication showed no to minimal change over the 2 years of treatment for subjects who remained on monotherapy. Average total daily doses at baseline and at 24 months are shown in Table 4.

**Table 4:** Summary of pharmacological and nonpharmacological treatment-by-visit and treatment

	Baseline		24 Months	
	Stimulant *N* = 305	Atomoxetine *N* = 395	Stimulant *N* = 244	Atomoxetine *N* = 274
Average total daily dose, mg/kg, mean (SD)	0.5 (0.2)^a^, 0.8 (0.3)^b^	1.0 (0.3)	0.5 (0.3)^a^, 0.9 (0.3)^b^	1.1 (0.3)
Average total daily dose, mg, mean (SD)	19.5 (9.7)^a^, 31.2 (12.1)^b^	37.7 (16.7)	20.3 (10.6)^a^, 32.2 (11.9)^b^	39.9 (17.0)
At least one nonpharmacological treatment, *n* (%)	91 (30.1)	156 (39.5)	88 (29.1)	158 (40.0)
Psychoeducation programs, *n* (%)	36 (11.9)	52 (13.2)	37 (12.3)	53 (13.4)
Counseling, *n* (%)	37 (12.3)	51 (12.9)	30 (9.9)	52 (13.2)
Cognitive behavioral therapy, *n* (%)	20 (6.6)	29 (7.3)	23 (7.6)	36 (9.1)
Family therapy, *n* (%)	2 (0.7)	11 (2.8)	2 (0.7)	17 (4.3)
Psychodynamic therapy, *n* (%)	1 (0.3)	3 (0.8)	7 (2.3)	10 (2.5)
Educational interventions in school, *n* (%)	21 (7.0)	24 (6.1)	21 (7.0)	35 (8.9)
Speech therapy, *n* (%)	7 (2.3)	20 (5.1)	7 (2.3)	26 (6.6)
Occupational therapy, *n* (%)	2 (0.7)	3 (0.8)	2 (0.7)	5 (1.3)
Relaxation techniques, *n* (%)	0 (0.0)	2 (0.5)	0 (0.0)	2 (0.5)
Hypnosis, *n* (%)	0 (0.0)	0 (0.0)	0 (0.0)	0 (0.0)
Psychomotor/physiotherapy, *n* (%)	6 (2.0)	13 (3.3)	7 (2.3)	10 (2.5)
EEG biofeedback, *n* (%)	1 (0.3)	0 (0.0)	0 (0.0)	0 (0.0)
Herbal/homeopathy, *n* (%)	0 (0.0)	2 (0.5)	0 (0.0)	1 (0.3)
Diet exclusion, *n* (%)	0 (0.0)	0 (0.0)	0 (0.0)	0 (0.0)
Diet supplement, *n* (%)	1 (0.3)	0 (0.0)	1 (0.3)	0 (0.0)
Other, *n* (%)	2 (0.7)	8 (2.0)	3 (1.0)	9 (2.3)

*EEG* electroencephalography, *n* number of affected subjects, *SD* standard deviation

^a^Methylphenidate

^b^Methylphenidate long-acting

**Fig. 2:**
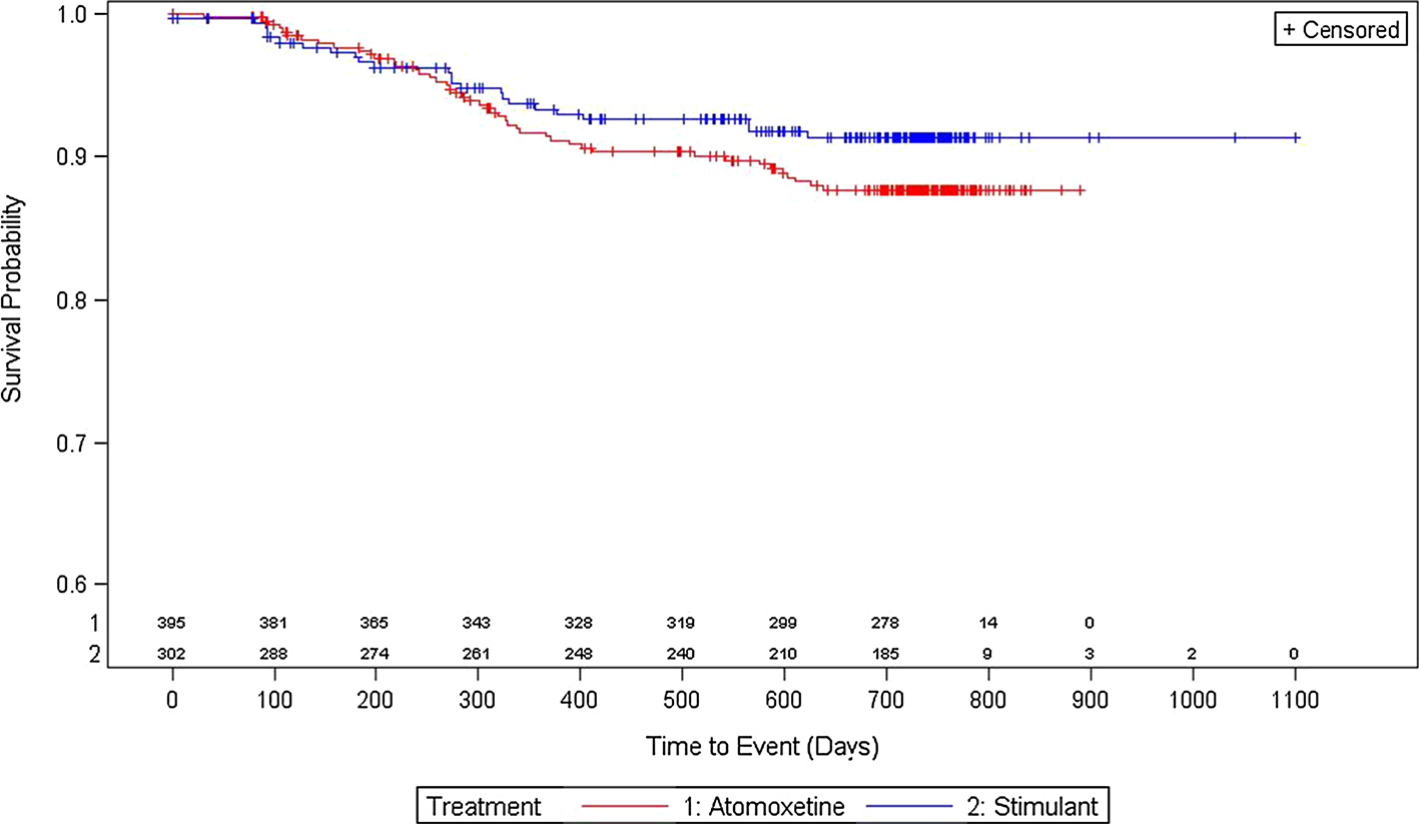
Kaplan–Meier plot for time to first change of therapy

**Fig. 3:**
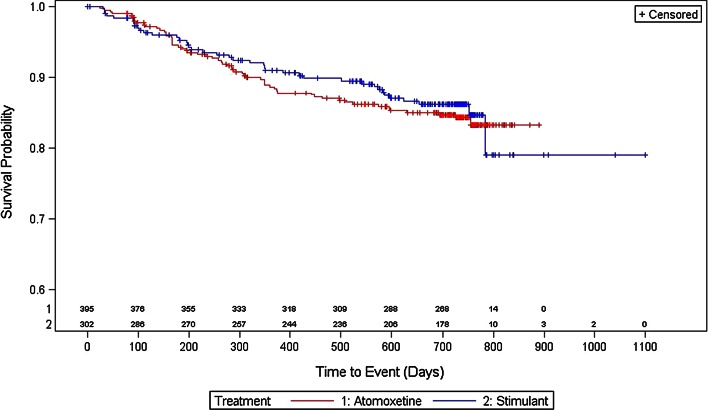
Kaplan–Meier plot for time to first treatment discontinuation

**Fig. 4:**
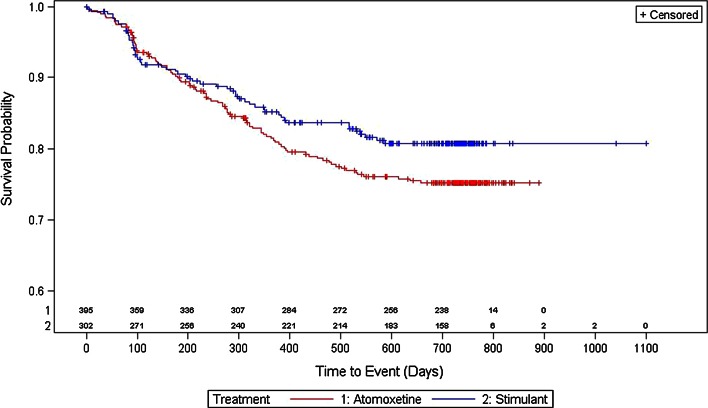
Kaplan–Meier plot for time to first dose change

Fewer subjects in the stimulant group at baseline changed therapy (7.9 vs. 11.4 %), discontinued treatment (13.2 vs. 14.9 %), or had a change of dose (17.9 vs. 23.3 %) versus subjects on atomoxetine at baseline. The log-rank test showed that there was no statistical difference between the treatments regarding time to any of these events. Time until 5 % of the population had an event is reported as the median time to event was not defined. As determined by the Kaplan–Meier survival curves, the estimated length in days and 95 % CI until 5 % of the population had an event for stimulant-treated versus atomoxetine-treated subjects for time to first change of therapy (Fig. 2) was 283.6 [153.8–565.3] days versus 269.6 [194.7–327.6] days, respectively. The estimated length in days and 95 % CI until 5 % of the population had an event for stimulant-treated versus atomoxetine-treated subjects for time to first treatment discontinuation (Fig. 3) was 194.7 [92.9–286.6] days versus 166.8 [113.9–244.7] days. The estimated length in days and 95 % CI until 5 % of the population had an event for stimulant-treated versus atomoxetine-treated subjects for time to first dose change (Fig. 4) was 89.9 [76.9–105.9] days versus 93.9 [81.9–126.8] days, respectively.

Few subjects (15 [2.1 %] at 24 months) switched to only receive nonpharmacotherapy during the study; however, over a third of subjects were receiving some form of nonpharmacotherapy at study entry. There was little change in nonpharmacological treatment participation over the course of 24 months (Table 4).

### Primary outcome measure

**Fig. 5:**
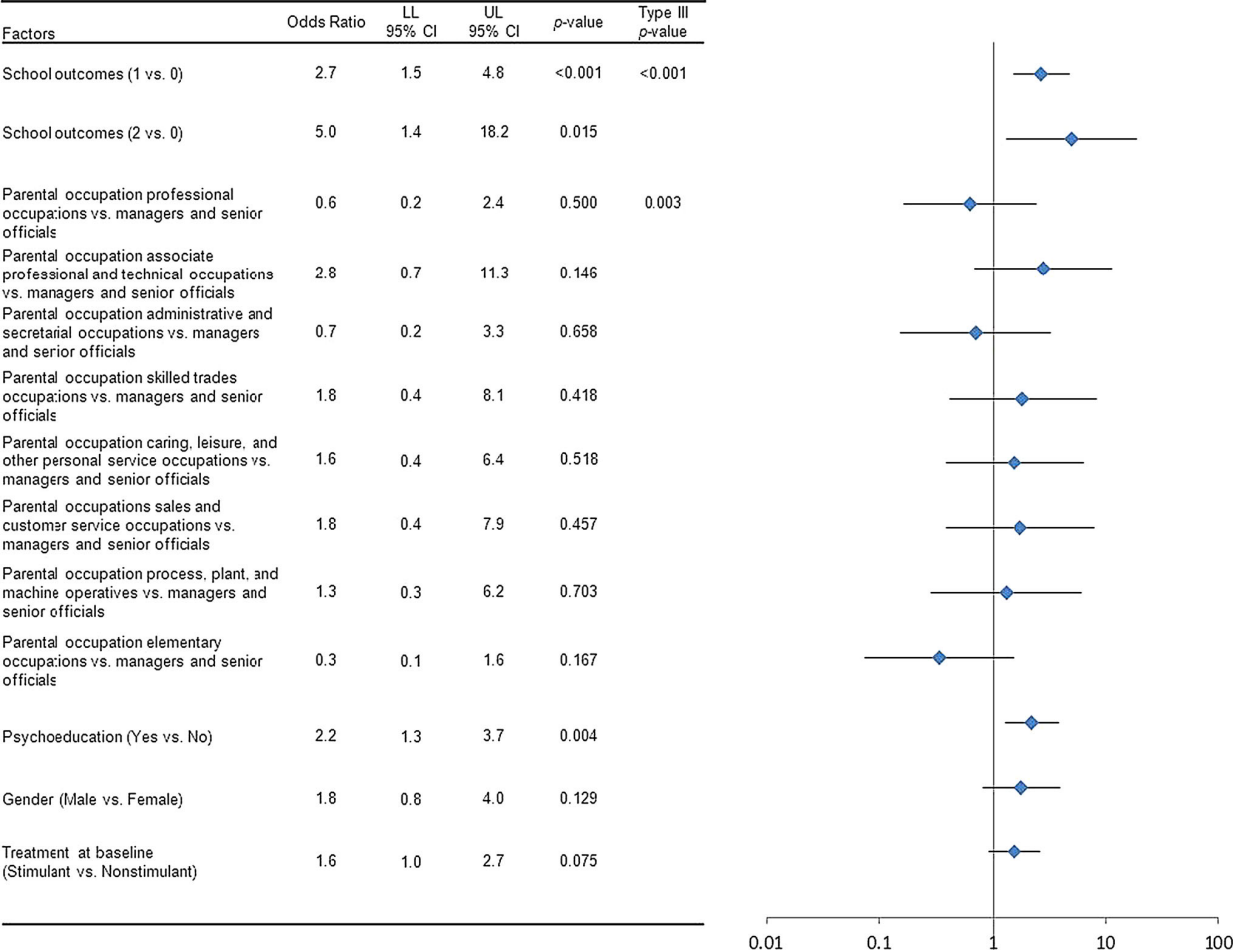
Factors associated with an increase in symptom severity in subjects with attention-deficit/hyperactivity disorder (from a multivariate logistic regression model). For each factor, adjusted odds ratios comparing each level against the baseline reference level and associated 95 % CI and Type III *p* values are presented. Additionally, for factors with more than 2 levels, corresponding homogeneity Type III *p* values are also shown. Estimates from the logistic regression model that included propensity scores and/or treatment compliance as time-varying covariates as additional adjusting factors were similar to the models without these additional adjustments. Abbreviations: *0* school outcome of manageable in a classroom environment; *1* school outcome of some exclusion from school lessons and/or in a special education program, *2* school outcome of not in school during the past 3 months, suspended from school, expelled from school, and/or requested to change to a special need school, *CI* confidence interval, *LL* lower limit, *UL* upper limit, *vs.* versus

Figure 5 presents the results from the multivariate logistic model estimated without PS and/or time-varying covariates. School outcome was statistically significant (Type III *p* < 0.001), with the odds of an increase in ADHD symptoms severity being significantly greater for subjects who had some exclusion from school lessons and/or were in a special education program than for subjects who were manageable in a classroom environment (odds ratio [95 % CI]; 2.7 [1.5–4.8]; *p* < 0.001). The odds of an increase in ADHD symptom severity were significantly greater in subjects who were not in school during the past 3 months, who were suspended from school, who were expelled from school, and/or who were requested to change to a special need school than in subjects who were manageable in a classroom environment at baseline (odds ratio [95 % CI]; 5.0 [1.4–18.2]; *p* = 0.015). Baseline parental occupation was also statistically significant (Type III *p* = 0.003) overall; however, none of the specific pairwise comparisons between parental occupations to the arbitrary reference group (managers and senior officials) were statistically significant. The odds of an increase in ADHD symptom severity were also significantly greater for subjects who received psychoeducation at baseline than for subjects who did not receive it at baseline (odds ratio [95 % CI]; 2.2 [1.3–3.7]; *p* = 0.004).

### Secondary outcome measures

For the secondary outcome measures, like the primary outcome analysis, the estimates from the logistic regression model including additional PS covariates and/or time-varying covariates were similar to the models without PS and without time-varying covariates. Therefore, results from the model without a PS and without time-varying covariates are reported. Factors associated with a decrease in ADHD symptom severity showed a statistically significant effect of parental work status (Type III *p* = 0.001), with the odds of a decrease in ADHD symptom severity being significantly greater for subjects who had parents who worked part-time at baseline than for those who had parents who worked for full-time pay at baseline (odds ratio [95 % CI]; 11.7 [3.4–39. 9]; *p* < 0.001).

**Fig. 6:**
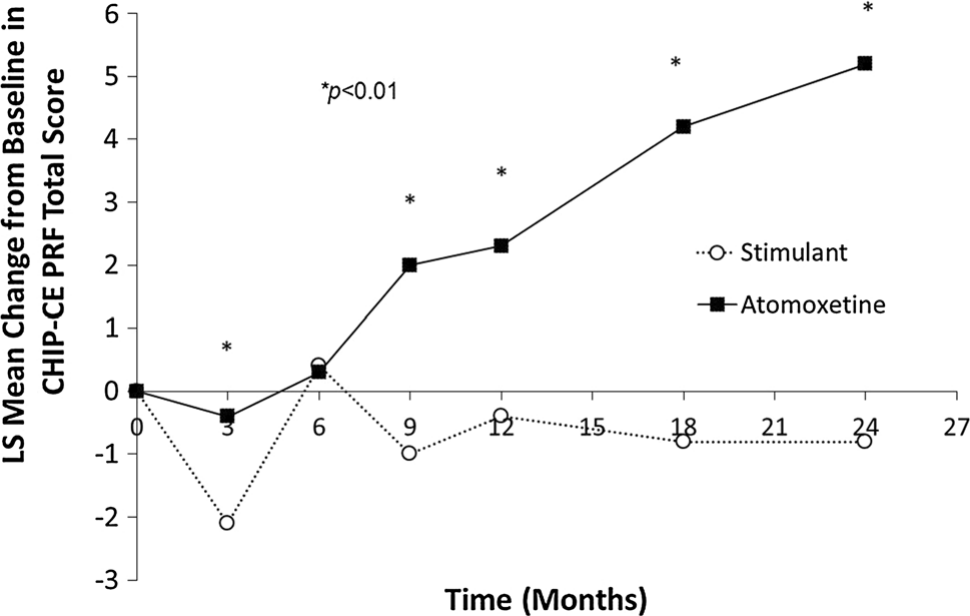
Least-squares mean change from baseline in the Child Health and Illness Profile, Child Edition-Parent Report Form total score from the longitudinal mixed-model repeated measures regression. Abbreviations: *CHIP-CE PRF* Child Health and Illness Profile-Child Edition, Parent Report Form, *LS* least squares

The MMRM analysis of changes from baseline in CHIP-CE PRF standardized total scores showed a statistically significant baseline treatment-by-visit interaction (Type III *p* < 0.001). At Months 3, 9, 12, 18, and 24, subjects who entered the study on stimulants had significantly less improvement from baseline in CHIP-CE PRF standardized total scores than subjects who entered the study on atomoxetine (Fig. 6). The estimates of the adjusted differences (least-square means) between subjects who entered on stimulants and atomoxetine were increasing over time. In particular, the maximum difference between baseline treatment groups was −6.0 (95 % CI −7.9, −4.1) at 24-month post-baseline.

Logistic regression models were used to study the association of different risk factors with each of the 4 protocol definitions of relapse (Table 5). The majority of subjects did not relapse at any time during the 2-year study. Higher baseline ADHDRS total score was associated with a decreased probability of relapse for the definitions that included ADHDRS total as a relapse criterion and an increased probability of relapse according to the definition based only on the CGI-ADHD-S. The odds of relapse were significantly greater for subjects who entered the study on stimulants, who had received psychoeducation at baseline, who had a family history of ADHD, or who had a comorbidity. The odds of relapse were significantly increased for subjects not manageable in a classroom environment.

**Table 5:** Factors associated with relapse and stability—odds ratios (95 % confidence intervals) and Type III *p* values from multivariate logistic regression models without propensity score adjustment or time-varying covariates included

Baseline treatment group	Relapse (4 definitions) OR (95 % CI)								Stability OR (95 % CI)	
	ADHDRS total increase ≥50 % and CGI–ADHD–S increase ≥2		ADHDRS total increase ≥50 %		CGI-ADHD-S ≥5		CGI-ADHD-S increase ≥2 at 2 or more consecutive post-baseline visits		CGI-ADHD-S ≤3 over the 2-year period	
	Stim	Atx	Stim	Atx	Stim	Atx	Stim	Atx	Stim	Atx
Relapsed/stable, %	4.0	3.3	19.9	13.2	8.6	4.6	8.6	4.1	79.0	85.1
Baseline ADHDRS total score	**0.9 (0.90, 0.98)** ^**a**^		**0.90 (0.88, 0.92)**		NI		**1.04 (1.01, 1.07)**		**0.98 (0.96–0.998**)	
Treatment at baseline (stimulant vs. atomoxetine)	1.7 (0.7, 3.9)		**2.1 (1.3, 3.3)**		1.8 (0.9, 3.7)		**2.4 (1.2, 4.8)**		**0.5 (0.3–0.8)**	
Gender (male vs. female)	NI		NI		NI		4.15 (0.97, 17.80)		0.6 (0.3, 1.1)	
Age	NI		NI		NI		2.8 (0.8, 9.4)		NI	
Psychoeducation at baseline (yes vs. no)	**3.0 (1.3, 7.0)**		NI		1.9 (0.9, 3.9)		**2.4 (1.2, 4.8)**		**0.6 (0.4–0.9)**	
School outcomes^b^										
Type III *p* value	NI		NI		*p* < 0.001		0.065		*p* < 0.001	
2 versus 0	NI		NI		**14.3 (3.4, 59.0)**		**4.5 (1.3, 16.2)**		0.5 (0.1, 1.9)	
1 versus 0	NI		NI		**5.1 (2.4, 10.8)**		1.2 (0.5, 2.5)		**0.4 (0.2–0.6)**	
Family history of ADHD										
Type III *p* value	NI		NI		0.134		NI		NI	
Immediate family versus none	NI		NI		**2.3 (1.1, 5.0)**		NI		NI	
Extended family versus none	NI		NI		2.7 (0.7, 10.7)		NI		NI	
Psychiatric comorbidity (yes vs. no)	NI		NI		**2.3 (1.1, 4.9)**		NI		0.7 (0.5, 1.1)	
Family setting at baseline (reference group is “other”)										
Type III *p* value	NI		0.066		NI		NI		NI	
Step parent	NI		6.6 (0.8, 56.5)		NI		NI		NI	
Single parent	NI		**9.7 (1.2, 79.2)**		NI		NI		NI	
Guardian	NI		2.3 (0.2, 24.3)		NI		NI		NI	
Separately with parents	NI		2.3 (0.2, 26.2)		NI		NI		NI	
Parents	NI		5.9 (0.8, 45.3)		NI		NI		NI	
Baseline parental occupation (reference group is “managers and senior officials”)									NI	
Type III *p* value	NI		NI		0.174		NI		<0.001	
Elementary occupations	NI		NI		0.3 (0.1, 2.1)		NI		3.04 (0.96, 9.58)	
Process, plant and machine operatives	NI		NI		0.5 (0.1, 4.1)		NI		1.4 (0.4, 5.1)	
Sales and customer service occupations	NI		NI		1.1 (0.2, 7.8)		NI		0.7 (0.2, 2.2)	
Caring, leisure, and other personal	NI		NI		0.9 (0.1, 5.6)		NI		0.8 (0.3, 2.3)	
Service occupations										
Skilled trades occupations	NI		NI		0.3 (0.03, 3.96)		NI		0.6 (0.2, 1.9)	
Administrative and secretarial occupations	NI		NI		0.7 (0.1, 4.3)		NI		1.0 (0.3, 3.2)	
Associate professional and technical occupations	NI		NI		2.2 (0.4, 12.4)		NI		0.4 (0.1, 1.3)	
Professional occupations	NI		NI		0.6 (0.1, 3.1)		NI		1.7 (0.6, 4.7)	

ADHD subtype, origin, substance use, total healthcare provider contacts, number of siblings, parental work status were not significantly associated with any definition of relapse or stability

*ADHD* attention-deficit/hyperactivity disorder, *ADHDRS* Attention-Deficit/Hyperactivity Disorder Rating Scale, *Atx* atomoxetine, *CGI*-*ADHD*-*S* Clinical Global Impression-Attention-Deficit/Hyperactivity Disorder-Severity, *CI* confidence interval, *NI* not included (variable not significant in the univariate model and not selected for inclusion in the full multivariate model; treatment was always included in the multivariate models, irrespective of being significant or not in the univariate models), *OR* odds ratio,* vs.* versus

^a^Bolded values are statistically significant (*p* < 0.05)

^b^School outcomes were as follows: 2 = if 1 of the following: not in school during the past 3 months, suspended from school, expelled from school, requested to change to a special need school; 1 = if 1 of the following: some exclusion from school lessons, in a special education program; 0 = if manageable in a classroom environment

Factors associated with stability (Table 5) showed a statistically significant effect of baseline school outcome (Type III *p* < 0.001), with the odds of stability being significantly lower for subjects who had some exclusion from school lessons or were in a special education program at baseline than for those who were manageable in a classroom environment at baseline. In addition, the odds of stability were significantly lower for subjects who entered the study on stimulants versus those who entered on atomoxetine and for subjects who received psychoeducation versus those who did not. The odds of stability decreased by a factor of 0.98 when the baseline ADHDRS total score increased 1 unit, keeping the other variables constant. There was a significant effect of baseline parental occupation in the multivariate model with or without time-varying covariates added (Type III *p* < 0.001), but the comparison of subjects who had parents in elementary occupations versus those whose parents were managers and senior officials was only statistically significant in the model with time-varying covariates added (odds ratio [95 % CI]; 4.2 [1.3–14.0]; *p* = 0.019). When the logistic regression model included PS, the results were generally similar to the logistic regression without PS with one exception: The odds of stability estimated with the model that included PS were significantly lower in subjects with at least one psychiatric comorbidity than in subjects without a psychiatric comorbidity (odds ratio [95 % CI]; 0.5 [0.3–0.9]; *p* = 0.014), whereas the odds ratio for stability was not significant in the model that did not include PS (Table 5).

For the GIPD total score, the adjusted difference between stimulants and atomoxetine averaged over the 2-year period was statistically significant (estimate [95 % CI]; 0.3 [0.1–0.4]; *p* < 0.001), with patients who entered the study on stimulants being more likely to have investigator-perceived difficulties (i.e., higher GIPD total scores) than patients who entered on atomoxetine at all post-baseline time points (Month 3 [LS mean change from baseline stimulant vs. atomoxetine; −0.1 vs. −0.3; *p* = 0.014]; Month 6 [−0.2 vs. −0.4; *p* = 0.048], Month 9 [−0.2 vs. −0.4; *p* = 0.006]; Month 12 [−0.1 vs. −0.4; *p* = 0.005]; Month 18 [−0.1 vs. −0.5; *p* < 0.001]; and Month 24 [−0.1 vs. −0.6; *p* < 0.001]). Similar results were observed for the estimated averaged difference between stimulants and atomoxetine when the other 3 GIPD questions were analyzed using the MMRM: “Difficulty during school” (Type III *p* < 0.001), “Difficulty during homework” (Type III *p* < 0.001), and “Difficulty over the entire day including night” (Type III *p* = 0.010). MMRM estimates for the treatment-by-visit interaction were not statistically significant for the 2 GIPD questions “Difficulty in morning” (Type III *p* = 0.411) and “Difficulty in evening” (Type III *p* = 0.971), indicating a constant difference between treatments at all visits during the 2-year period. The estimated average treatment effect for each of these GIPD questions showed that patients who entered the study on stimulants were more likely to have investigator-perceived difficulties in the morning (estimate [95 % CI]; 0.3 [0.2–0.5]; *p* < 0.001) and in the evening (0.3 [0.1–0.4]; *p* < 0.001) than patients who entered on atomoxetine.

### Tolerability

Overall, the percentages of solicited AEs were low and generally decreased throughout the course of the study for subjects who entered the study on stimulants or atomoxetine (Table 6).

**Table 6:** Percentage of solicited adverse events that interfered with subjects’ functioning or health-related quality of life

Time point Tolerability variable	Overall^a^ *N* (% [95 % CI])	Stimulant at baseline *N* (% [95 % CI])	Atomoxetine at baseline *N* (% [95 % CI])
Baseline	*N* = 704	*N* = 302	*N* = 395
Abdominal pain	6 (0.9 [0.3–1.8])	3 (1.0 [0.2–2.9])	3 (0.8 [0.2–2.2])
Changes in personality	2 (0.3 [0.0–1.0])	2 (0.7 [0.1–2.4])	0
Decreased appetite	27 (3.8 [2.5–5.5])	7 (2.3 [0.9–4.7])	20 (5.1 [3.1–7.7])
Fatigue	5 (0.7 [0.2–1.6])	1 (0.3 [0.0–1.8])	4 (1.0 [0.3–2.6])
Headaches	9 (1.3 [0.6–2.4])	3 (1.0 [0.2–2.9])	6 (1.5 [0.6–3.3])
Insomnia	14 (2.0 [1.1–3.3])	4 (1.3 [0.4–3.4])	10 (2.5 [1.2–4.6])
Sleepiness	3 (0.4 [0.1–1.2])	2 (0.7 [0.1–2.4])	1 (0.3 [0.0–1.4])
Month 12	*N* = 607	*N* = 252	*N* = 348
Abdominal pain	4 (0.7 [0.2–1.7])	1 (0.4 [0.0–2.2])	3 (0.9 [0.2–2.5])
Changes in personality	3 (0.5 [0.1–1.4])	1 (0.4 [0.0–2.2])	2 (0.6 [0.1–2.1])
Decreased appetite	11 (1.8 [0.9–3.2])	3 (1.2 [0.2–3.4])	8 (2.3 [1.0–4.5])
Fatigue	0	0	0
Headaches	9 (1.5 [0.7–2.8])	2 (0.8 [0.1–2.8])	7 (2.0 [0.8–4.1])
Insomnia	9 (1.5 [0.7–2.8])	5 (2.0 [0.6–4.6])	4 (1.1 [0.3–2.9])
Sleepiness	0	0	0
Month 24	*N* = 559	*N* = 222	*N* = 331
Abdominal pains	1 (0.2 [0.0–1.0])	0	1 (0.3 [0.0–1.7])
Changes in personality	1 (0.2 [0.0–1.0])	1 (0.5 [0.0–2.5])	0.0
Decreased appetite	3 (0.5 [0.1–1.6])	1 (0.5 [0.0–2.5])	2 (0.6 [0.1–2.2])
Fatigue	0	0	0
Headaches	2 (0.4 [0.0–1.3])	1 (0.5 [0.0–2.5])	1 (0.3 [0.0–1.7])
Insomnia	4 (0.7 [0.2–1.8])	2 (0.9 [0.1–3.2])	2 (0.6 [0.1–2.2])
Sleepiness	0	0	0

*CI* confidence interval, *MPH* methylphenidate, *N* number of subjects

^a^No solicited adverse events that interfered with subjects functioning or health-related quality of life were reported for treatment categories other pharmacotherapies (*N* = 5 [60 % antipsychotics and 40 % other), or a drug combination (*N* = 2 [100 % MPH long-acting and atomoxetine])

The GEE logistic regression analysis indicated that the treatment-by-visit interactions for abdominal pain, fatigue, and headache were not statistically significant. The estimates of the odds ratios averaged over all visits indicated that patients who received stimulants at baseline were less likely to experience abdominal pain (averaged odds ratio [95 % CI]; 0.5 [0.3–0.7]; *p* = 0.002); fatigue (averaged odds ratio [95 % CI]; 0.4 [0.2–0.9]; *p* = 0.018); and headache (averaged odds ratio [95 % CI]; 0.4 [0.3–0.7]; *p* < 0.001). The baseline treatment-by-visit interactions were statistically significant for decreased appetite (Type III *p* = 0.05) and insomnia (Type III *p* = 0.017). For decreased appetite, there was a statistical difference in the odds of having decreased appetite only at the 24-month visit, with greater odds for subjects receiving stimulants at baseline (odds ratio [95 % CI]; 3.3 [1.5–7.1]; *p* = 0.002). For insomnia, a statistically significant difference was observed between baseline treatment groups only at Month 12 with the odds of having insomnia being significantly lower in the group who received stimulants at baseline (odds ratio [95 % CI]; 0.5 [0.3–0.98]; *p* = 0.044).

## Discussion

This study characterizes factors associated with an increase in ADHD symptom severity during a 2-year follow-up period in subjects who were responders and stable on their first pharmacotherapy. In contrast to the magnitude of switching observed in the ADORE study (Preuss et al. 2006), stability continued for the vast majority of subjects who were stable on their ADHD treatment for 3–8 months. Baseline treatments were maintained, there was minimal switching or changes in dose, and compliance was good. This difference might be due to ADORE being a study of patients newly initiated to treatment, while AUTOR was a study of treatment responders.

School outcome and parental occupation at baseline were identified as factors associated with an increase in ADHD symptom severity. Symptom severity was more likely to increase in subjects with a negative school outcome at baseline (vs. less negative school outcome). The effect of baseline parental occupation on ADHD symptom severity was less clear.

Psychoeducation at baseline was identified as a factor associated with an increase in ADHD symptom severity, which may be due to unmeasured confounding factors (i.e., variability in administration of psychoeducation sessions). Conversely, this outcome could be related to an earlier onset and to persistent ADHD symptoms, as European guidelines recommend beginning ADHD treatment with nonpharmacotherapy before initiating pharmacotherapy treatment. Similarly, ADORE investigators found that subjects initiated on psychotherapy and those who added psychotherapy to existing pharmacotherapy had a significant worsening of symptoms; this effect was most evident for psychoeducation counseling. Psychoeducation may have a deleterious effect if administered prior to a subject being stabilized on pharmacotherapy (Falissard et al. 2010). Details about the type of psychoeducation were not captured in this study, and the relative proportion of patients who received psychoeducation was small; future studies are needed to clarify this finding.

A secondary analysis identified parental work status at baseline as a significant factor, with symptom severity being more likely to improve in subjects whose parents worked part-time; this could reflect these parents having greater ability to provide additional support to ensure medication compliance and be involved in nonpharmacotherapeutic interventions.

When factors associated with the most stringent criteria of relapse (i.e., an increase of 50 % or greater on the ADHDRS total score and an increase in the CGI-ADHD-S score of at least 2 points) were examined, relapse was more likely to occur in subjects who received psychoeducation and subjects with higher baseline ADHDRS total scores. These two factors were most consistently associated with relapse, regardless of the definition. Worse baseline school outcomes, prior family history of ADHD, and presence of certain psychiatric comorbidities were associated with relapse only when it was measured based on the CGI-ADHD-S. Analysis of factors associated with stability showed that subjects who entered the study on stimulants were less likely to maintain their initial response than those who entered the study on atomoxetine; subjects with higher baseline ADHDRS total scores were less likely to maintain their initial response. The overall relapse rates were much lower than in the initial phase of the relapse prevention trial of atomoxetine (Michelson et al. 2004), possibly due to the longer response period required for entry into the AUTOR study. The observed relapse rates during the continuation period of the relapse prevention trial of atomoxetine (Buitelaar et al. 2007) are comparable to those observed in AUTOR.

Additional secondary analyses showed that subjects who entered the study on stimulants reported significantly lower QoL, as measured by the CHIP-CE PRF than subjects who entered the study on atomoxetine over 2 years. This difference was most noted in the satisfaction and comfort domains. Among treatment-naïve patients randomized to treatment with atomoxetine versus other ADHD pharmacotherapy (comprised mostly of patients taking methylphenidate), atomoxetine-treated patients had significantly lower improvement on the CHIP-CE achievement domain at 6 months, but there was no significant difference between treatments at 12 months in this domain (Fuentes et al. 2013). The CHIP-CE total score was not computed by Fuentes et al. The difference in their finding of improvement in CHIP-CE domain scores for the other ADHD pharmacotherapy may be due to a difference in study population, as they examined patients who were naïve to treatment and as our study was comprised of 3- to 8-month treatment responders. The continued improvement in QoL for patients initiated on atomoxetine differs slightly from the findings of the relapse prevention trial (Michelson et al. 2004) in which this outcome was measured with the Child Health Questionnaire. These investigators found that under blinded conditions, maintenance of treatment with atomoxetine was associated with significantly less worsening of QoL than was removal of treatment.

Subjects who entered as responders to stimulants had greater investigator-perceived difficulties in the morning, during school, during homework, over the entire day, and in the evening as measured by the GIPD when compared with subjects who entered as responders to atomoxetine.

### Limitations

As an observational trial, subjects were not randomized to treatment, and treatment decisions were left to the investigator and subject; thus, treatment comparisons are subject to bias and confounding. Propensity scores were used to adjust for the probability of receiving one treatment or another, depending on differences in subject baseline characteristics; however, differences between treatment groups cannot be considered causal.

The study enrolled subjects who had responded to an initial 3–8 months of treatment with ADHD medication; therefore, the results generalize to that population rather than to all treated ADHD subjects. Additionally, patients were recruited within practices where they were treated; thus, the physician population reflects the real-world treatment patterns for ADHD in these European countries. The majority of subjects were recruited from Italy, Romania, and Greece. and the pattern of allocation to treatment reflects the timing of medication availability in those regions; therefore, factors associated with ADHD worsening are driven by the cultural, social, and economic factors of those countries during that period.

The AEs conclusions are limited because subjects had been on the same therapy for 3–8 months, which led to a lower rate of AEs overall than would be expected in patients who initiated on pharmacotherapy. Additionally, information was solicited only for specific events common with these treatments. AUTOR was not designed to characterize long-term tolerability with these medications.

In conclusion, in this observational study of more than 700 European children and adolescents with ADHD who were 3- to 8-month responders to their first pharmacotherapy, worsening of symptoms was associated with the initial use of psychoeducation, parental occupation, and poorer school outcomes, but not to initial treatment administered; however, having achieved treatment response for a 3- to 8-month period on atomoxetine was associated with improved QoL and ADHD symptom stability. AUTOR extends the ADHD relapse prevention studies by characterizing the performance of pharmacotherapy for responders in a naturalistic setting.
